# A perspective on neural and cognitive mechanisms of error commission

**DOI:** 10.3389/fnbeh.2015.00050

**Published:** 2015-03-03

**Authors:** Sven Hoffmann, Christian Beste

**Affiliations:** ^1^Performance Psychology, Institute of Psychology, German Sport University CologneCologne, Germany; ^2^Cognitive Neurophysiology, Department of Child and Adolescent Psychiatry, Faculty of Medicine of the TU Dresden, University Hospital Carl Gustav CarusDresden, Germany

**Keywords:** action selection, performance monitoring, basal ganglia, dopamine function, dual-process theory of dopamine function, biased competition, error processing, dopamine

## Abstract

Behavioral adaptation and cognitive control are crucial for goal-reaching behaviors. Every creature is ubiquitously faced with choices between behavioral alternatives. Common sense suggests that errors are an important source of information in the regulation of such processes. Several theories exist regarding cognitive control and the processing of undesired outcomes. However, most of these models focus on the consequences of an error, and less attention has been paid to the mechanisms that underlie the commissioning of an error. In this article, we present an integrative review of neuro-cognitive models that detail the determinants of the occurrence of response errors. The factors that may determine the likelihood of committing errors are likely related to the stability of task-representations in prefrontal networks, attentional selection mechanisms and mechanisms of action selection in basal ganglia circuits. An important conclusion is that the likelihood of committing an error is not stable over time but rather changes depending on the interplay of different functional neuro-anatomical and neuro-biological systems. We describe factors that might determine the time-course of cognitive control and the need to adapt behavior following response errors. Finally, we outline the mechanisms that may proof useful for predicting the outcomes of cognitive control and the emergence of response errors in future research.

## Introduction

Errare humanum est, sed in errare perseverare diabolicum (Seneca). In other words: “who commits an error and does not correct it, commits a second one” (Confucius). Similar notions can be found in texts by Seneca, Horaz, Cicero and Aristotle. Already these philosophical notions stipulate the relevance and importance of the detection and compensation of errors. However, obviously there exist several types of errors. Basically, one can commit “mistakes” (e.g., not knowing the correct decision) or “slips” (the selected action is not what has been intended). The latter is what is this manuscript is about: a situation leading to an inappropriate action selection, likely making you think: “Upps.”

We constantly evaluate our own actions, and such evaluations are important for goal-directed behavior. This type of evaluation marks the endpoint of the solving of one of the main problems every creature is constantly confronted with: the choice between behavioral alternatives or between competing systems that seek simultaneous access to a restricted resource (Mink, 1996; Redgrave et al., 1999). Action selection is clearly error prone, meaning that we do not always select the appropriate action in a given situation. Rather, we must use these errors to adapt our behavior to changes in environmental demands. This process requires constant monitoring, evaluation and adaptation of one's own actions in accordance with environmental demands.

However, many research and theories (refer Boxes 1, 2) focus on the processes and modulators of processes that follow an error, or its consequences from a more cognitive or computational perspective, but do not address the processes that precede an error as well as detail possible neurobiological and functional neuroanatomical aspects possibly important to understand how errors are committed. Similarly, the research in the fields of response control and error monitoring reviewed above is committed to identifying the determinants of error processing and not the determinants of “error commissioning.” There are a few noteworthy exceptions in literature, as the work by Ridderinkhof et al. (2003) examining EEG correlates of processes occurring before an error, or the work by Cavanagh et al. (2011, 2012) showing that distinct neural oscillations in the theta frequency band reflect processes of the fore-period of an error, or the work by Weissman et al. (2006) pointing to the importance of attentional processes in this regard. “Error commissioning,” though common to everybody as a phenomenon of importance in daily life, is still not in the focus of the cognitive neuroscience community, especially if compared to the vast amount of research conducted in the last decades on the consequences of an error and their neuronal mechanisms. Therefore, in the current review we will outline what theoretical concepts may be important to consider when trying to examine the mechanisms determining the commission of errors.

Box 1 **Theories of error processing.**

Historically, the “mismatch hypothesis” is an early hypothesis that assumes that the neural representations of initiated and demanded (re-)actions are compared, which means that the error signal (reflected in the Ne/ERN) reflects a process that compares the output of the motor system (i.e., an efference copy) with the plan of the response (Falkenstein et al., 1990; Gehring et al., 1993; Scheffers et al., 1996). However, today the most influential models seem to be: (i) the conflict model, (ii) the reinforcement learning (RFL) hypothesis, and possibly (iii) the predicted-response outcome (PRO) model.

The conflict monitoring theory (Carter et al., 1998; Botvinick et al., 2001; van Veen and Carter, 2002) assumes that in all situations in which two or more actions can be performed, a conflict between these response options emerges. The term conflict refers to a temporal overlap of (pre-) activated response sets. This conflict signals the need to increase control. Errors (at least fast guesses) emerge from conflicts of nearly simultaneously established response representations wherein the erroneous response is nearly automatically activated (e.g., Yeung et al., 2004). However, conflict can even exist at the attentional and stimulus-processing levels, at least in situations that require efficient perceptual processing.

The reinforcement learning hypothesis (RFL; Holroyd and Coles, 2002) assumes that error signals are carried by the mesencephalic dopamine system and are used to train the ACC to optimize performance on the task at hand. In this theory, the ACC acts as a motor control filter that decides which motor commands are issued to the motor system. The theory of Holroyd and Coles (2002) details how an error signal is generated by the ACC and is based on the notion that the major mechanism by which errors are detected relies upon the temporal difference model (TD-model) of dopamine function (Suri and Schultz, 1998, 2001; for review see Suri, 2002). The RFL is not at odds with conflict theory. Holroyd et al. (2005) suggested integration of the conflict model and RFL.

The third model is the predicted-response outcome (PRO) model (Alexander and Brown, 2011). This model focuses on the functional role of the medial PFC, or more specifically, the ACC, with respect to errors, error likelihood, conflict, reward valence, and punishment. This model is a probabilistic model of the medial PFC. It assumes that the medial PFC is mainly involved in learning and predicting the outcome of actions, regardless of the motivational saliency. The model assumes that during the time course of an experiment, the ACC learns a timed prediction of the possible responses that are related to a stimulus and the corresponding outcomes. The signal from which this relationship is learned consists of integration of response-outcome combinations, i.e., prediction errors, which consist of unexpected outcomes and unexpected non-occurrences (cf. Alexander and Brown, 2011). The PRO model suggests the mPFC to be not only the key region with respect to cognitive control, but also that the mPFC is concerned with and establishing predictions with respect to actions in general.

However, it needs to be noted that all of these models assume that individual become more accurate after an error, an effect that is related to the post-error slowing effect (i.e., a reduction in the speed of responding after an error). However, several lines of evidence suggest that these effects only occur when error are infrequent (Notebaert et al., 2009) and is also modulated by the instruction (i.e., focus on accuracy vs. speed, Jentzsch and Leuthold, 2005) as well as if the task is self-paced (Steinborn et al., 2012).

Box 2 **Properties of the error processing signal.**

In the electroencephalogram (EEG), an error is reflected by a negative deflection (at approximately 60 ms) at fronto-central electrode positions. This deflection is known as the error negativity (Ne, Falkenstein et al., 1990) or error-related negativity (ERN, Gehring et al., 1993). However, even following correct responses, a similar negativity (correct-response negativity, CRN) can be observed (Vidal et al., 2000, 2003; Hoffmann and Falkenstein, 2010). Some evidence exists that suggests that the CRN and Ne/ERN are reflections of the same neural system (Hoffmann and Falkenstein, 2010; Roger et al., 2010) that is central to the adaptation of actions. The modality does not modulate the ERN (e.g., Masaki et al., 2001; Endrass et al., 2005) or CRN (Falkenstein et al., 1991; Forster and Pavone, 2008) and the occurrence is not restricted to choice reaction tasks (Falkenstein et al., 1995; Gehring et al., 1995; Gehring and Willoughby, 2002; Hoffmann and Falkenstein, 2011, 2012; Hoffmann and Wascher, 2012).

Several studies have demonstrated the involvement of the rostral cingulate cortex (rACC; Kiehl et al., 2000; Mathalon et al., 2003; Klein et al., 2007a) and the pre-supplemental motor area (pre-SMA; Ridderinkhof et al., 2004). Specifically, the importance of the anterior cingulate cortex (ACC) has been supported by several findings based on functional imaging. An error-related blood oxygen level-dependent (BOLD) signal increase in the ACC has been found (Menon et al., 2001; Ridderinkhof et al., 2004; Taylor et al., 2007) and has been shown to be predictive of the strength of the Ne/ERN measured via EEG (Dehaene et al., 1994; Ullsperger and von Cramon, 2001; Debener et al., 2005; Willemssen et al., 2011; Beste et al., 2012b; Hoffmann et al., 2014). The importance of the ACC for error processing is also corroborated by single-unit recordings in rodents (Stuphorn et al., 2000; Emeric et al., 2010) and lesion studies (Swick and Turken, 2002). In addition to the ACC, the anterior insular cortex (AIC) has been demonstrated to be involved in error processing (Ullsperger and von Cramon, 2003; Hester et al., 2004; Klein et al., 2007a; Ullsperger et al., 2010). However, aside these neocortical structures also the basal ganglia play an important role (Falkenstein et al., 2001; Beste et al., 2006, 2009; Ito and Kitagawa, 2006; Beste et al., 2007, 2008; Willemssen et al., 2008). The importance of the basal ganglia for error monitoring and error-related behavioral adaptation may at least partly relate to the importance of the dopaminergic for error processing (refer Box 1), which is suggested by several studies on psychiatric diseases affecting the dopamine system (Mathalon et al., 2002; Ridderinkhof et al., 2002; Holroyd and Yeung, 2003; Easdon et al., 2005; Liotti et al., 2005), and foremost by neuropharmacological studies (e.g., Zirnheld et al., 2004; de Bruijn et al., 2006; Willemssen et al., 2009) and neurogenetic studies (Frank et al., 2007; Klein et al., 2007b; Krämer et al., 2007). However because the dopaminergic system strongly interacts with other neurotransmitter systems, the monitoring of errors is also modulated by those neurotransmitter systems (Tieges et al., 2004; Baune et al., 2010; Beste et al., 2010a,b,c, 2011a, 2013). For a detailed review of the neurobiological factors (i.e., neurotransmitter systems) that influence performance monitoring processes, see Jocham and Ullsperger (2009).

“Error commissioning” may be defined as misguided action/response selection, i.e., a failure in the multitude of selection processes that occur prior to a motor response. However, besides these top–down mechanisms, also other factors related to the bottom–up processing of stimuli need to be considered. Starting from a simple example we will outline which components of different theoretical conceptions may be useful to derive future formal models of error commission. The review will conclude with a perspective on the venues of future research and approaches that may be combined to approach the mechanisms underlying error commissions.

## A cognitive view on error commission

Before outlining the possible neurophysiological mechanisms that are probably important to consider when one is interested in the neural mechanisms underlying the commission of errors, the question of how the involved constructs, such as decisions, action selection and erroneous and correct information processing can be quantified to relate these contracts to the described neurophysiological mechanisms arises. In a wider sense, the described mechanisms, i.e., information processing or action selection, can be defined as the processes that are involved in decision making.

In the field of cognitive psychology, a vast literature on well-established models related to this topic exists. Thus, a review in this regard will always be selective. However, it is helpful, to start with a how errors emerge from a cognitive perspective.

Basically, errors might emerge due to deficient knowledge (i.e., mistakes); but also due to deficient vigilant attention (for a detailed review cf. Langner and Eickhoff, 2013), due to inappropriate stimulus processing, or due to deficient response selection, i.e., response conflict. Another view is that there are basically two error types: impulsive errors (i.e., slips, fast guessing), and errors due to cognitive overload (Reason, 1990). Thus, in experimental designs, errors are due to quite different manipulations. It would go far beyond the scope of the present review to present ALL possibilities in this regard. However, a key factor that provokes errors is task difficulty, which is closely related to cognitive efficiency such that performance in a task at hand is a function of speed and accuracy, or speed accuracy trade-off (SAT). Indeed, error-likelihood does not vary only on a purely random way. Already Rabbitt (1966) found that subjects adjust their strategy immediately following errors in order to adapt on a behavioral level. Later, it was found that subjects set up response criteria a priori in order to adapt their response strategy with respect to task difficulty (this was termed Macro-SAT), but they also adapt on a single-trial level due to carry over effects of one trial to another (i.e., Micro-SAT; Jentzsch and Leuthold, 2005; Jentzsch and Dudschig, 2009). Furthermore, subjects can proactively adjust their strategy, i.e., response threshold in order to prevent errors (Brown and Braver, 2005) if a valid cue is provided to the participants.

Coming back to the different types of errors, it has to be stipulated, that in the present review we focus on errors due to fast guesses or impulsive errors due to e.g., misallocation of attention. Indeed, the mechanisms described herein might be quite different in other error types due to the involved cognitive mechanisms. Already Gehring et al. (1993) suggested that impulsive errors induce post-error slowing which is completely different in errors due to cognitive overload: here, there is no post-error slowing observable (Hochman and Meiran, 2005). Going more into detail with respect to the causes of errors, it was found that obviously distracting or even aversive information might play a crucial role: e.g., auditory noise affects errors rate considerably (Steinborn and Langner, 2011). Another point is, that the number of alternative responses is closely related to error probability (Hick, 1952). This is due to the fact, that guessing in a situation with many response alternatives is not quite a good idea, since almost any lapse of attention or slip will result in an error because the basic probability of being correct by guessing is quite low in this situation. Highly relevant in the context of the present review is that the key correlate of response, or error monitoring is correlated with the number of response alternatives (Maier et al., 2010) indicating that increasing the number of response alternative decreases or negatively affects response monitoring mechanisms. This might well play a role with respect to adaptive strategies throughout the experiment and thus error commission.

But how can one model the processes involved in stimulus processing and response selection? One model, the dual-stage model (DSTP; Hübner et al., 2010) assumes two phases (an early and a late phase) of response selection (Hübner et al., 2010). During the first phase, stimulus information affects the categorical selection and response selection processes, and during the second phase, response selection is driven by categorical selection. However, an open debate exists (e.g., White et al., 2011) with respect to the question whether this model can be generalized, since it was derived from a flanker task. Anyway, different error types induce different strategies and thus likely different neural structures or functions are involved. This is also important in the context of the cognitive model with respect to binary decision we focus on in the following. What is interesting in this model is that it might not only be capable to model both errors types, it is also appealing, since it can be linked to neural models as well.

**Figure 1:**
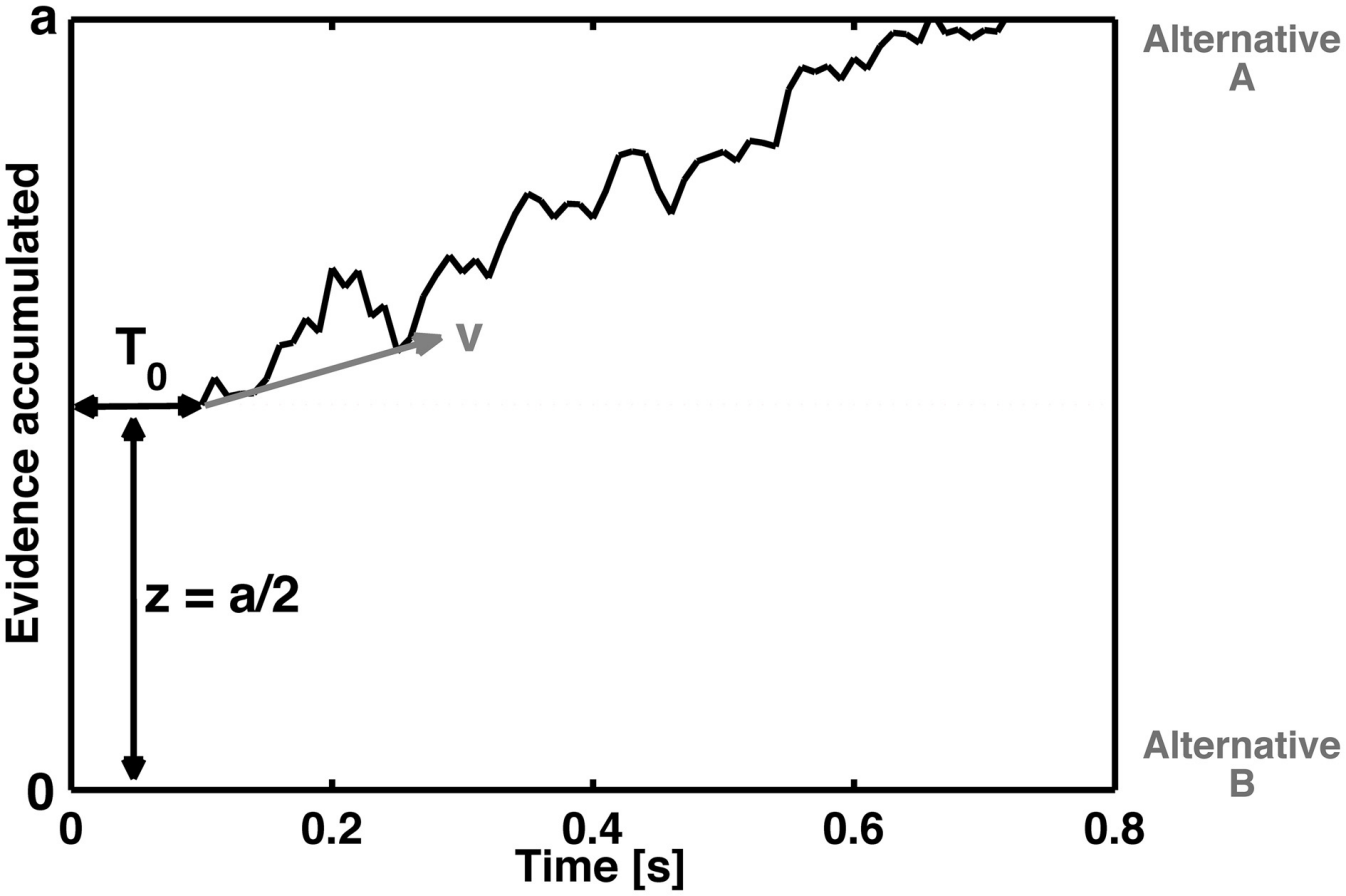
**Schematic representation of the diffusion process of a single decision between two response alternatives A and B**. The curved line represents the accumulation of information over time until boundary separation (*a*) is reached, which is the time point of the decision for the corresponding response. The reaction time is therefore a function of the boundary separation, the speed of information processing (as reflected by the steepness of *z*), the non-decisional time *T*_0_ (which reflects basic stimulus processing) and the starting point (*z*) (which refers to how conservative or liberal the subject is with respect to error commissioning or to one of the two response alternatives).

In the cognitive modeling of decision-making, it is assumed that evidence related to stimulus processing is accumulated via a stochastic, noisy process until a decision criterion is reached (e.g., Ratcliff, 2013). The drift diffusion model (DDM, Ratcliff, 1978) decomposes reaction time distributions and error rates into several parameters. Basically, the DDM assumes an accumulation of information during the performance of a binary choice. This information accumulation is described as a stochastic process that drifts into different decisional outcomes, for example left or right, or, in the context of the present manuscript, correct or incorrect choices (see Figure 1).

The four basic parameters of the DDM (Figure 1) are the drift rate (*v*), boundary or threshold separation (*a*), starting point (*z*), and the duration of the non-decisional process (*T*_0_). In terms of cognitive psychology, the drift rate represents the speed of information accumulation or the speed of information processing. Thus, the drift rate is a performance measure that reflects, for example, the difficulty of the task. Smaller drift rates are associated with more difficult tasks. The boundary separation (*a*) describes how much information is needed for a decision. Large values indicate rather conservative decision strategies, and small values indicate rather liberal decision strategies. Indeed, the boundary separation is sensitive to speed and accuracy instructions (Voss et al., 2004). The starting point (*z*) describes the a priori bias associated with one of the choices. Such a bias could arise if, for example, one of the responses is associated with a larger reward than the other (Voss et al., 2004). Moreover, trait-like biases can influence the starting point; e.g., obsessive–compulsive disorder patients tend to increase their response monitoring, as indicated by the ERN (Endrass et al., 2008; Hajcak et al., 2008; Endrass et al., 2010; Mathews et al., 2012). The fourth parameter, i.e., the duration of non-decisional processes, quantifies processes such as basic encoding processes and/or the process of response execution (more specifically, motor activity). In summary, these parameters are closely related to the question of how the time course and cognitive processes involved in error commissioning can be quantified and predicted.

## A neuro-cognitive perspective of error commissioning

Let's first consider an example to describe and to integrate how different existing theoretical conceptions may be combined to understand the mechanisms that lead to error commissioning. After this description, we will examine the evidence and conceptual grounds for a neuro-cognitive perspective in detail (refer section A Neuro-cognitive Perspective of Error Commissioning). Along the lines of this example, we will elucidate different conceptions and the links between these conceptions to understand the mechanisms of error commissioning and how these may influence future formalized computational models of errors commission. Goal of this integrative review is to provide an overview of potential neurobiological and functional neuroanatomical factors that are possibly essential to consider when interested in the neural mechanisms leading to an error. Therefore, we do not intend to put forward a formalized computational model of error commissioning.

**Figure 2:**
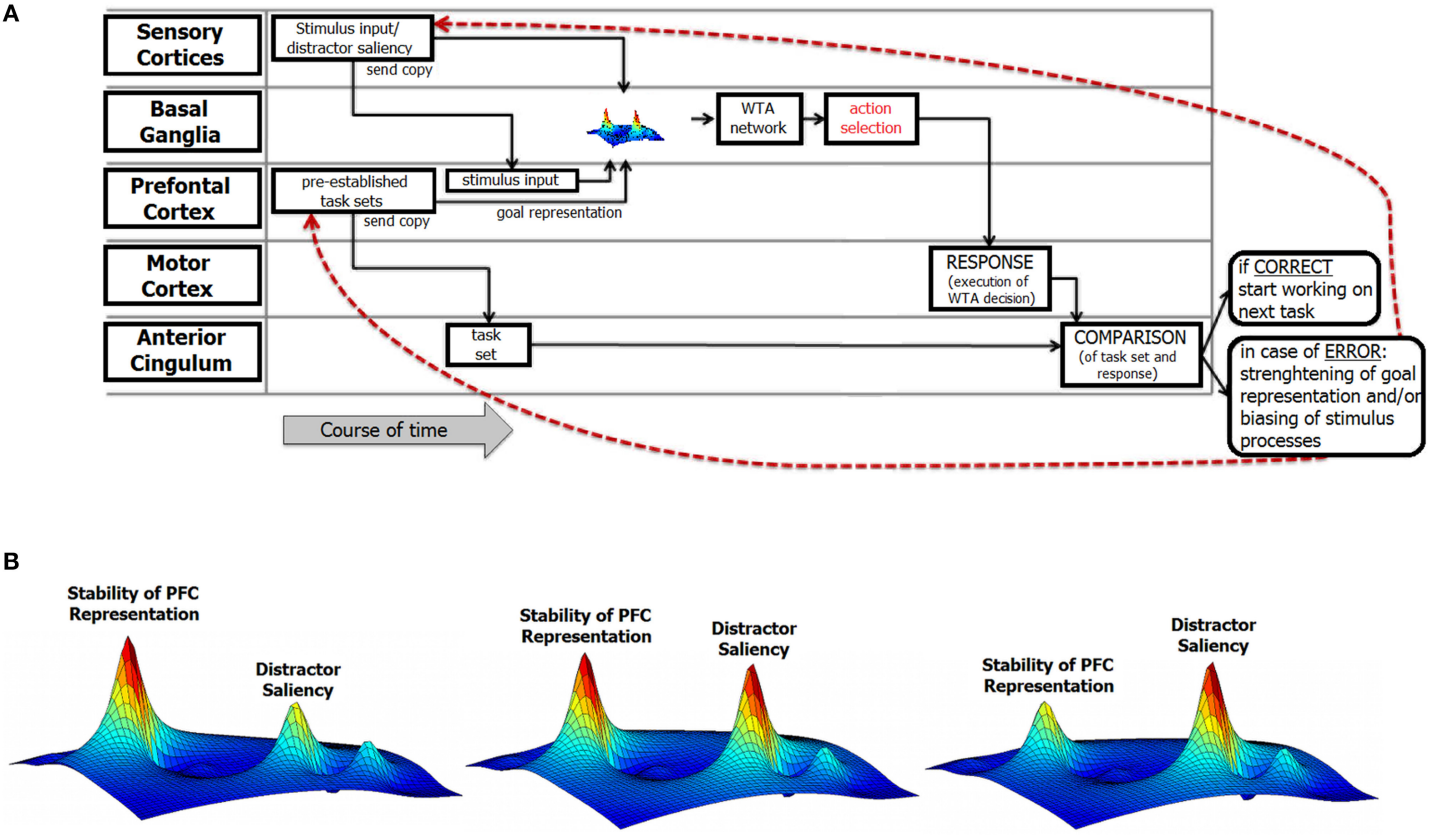
**Illustration of the structure of the integrative review. (A)** This figure illustrates the functional components of error processing and includes information about their relative relevance in the error monitoring process over time and their corresponding theoretical links. At the left of the figure, the different theoretical conceptions are outlined. Each of these theories refers to either one specific or various functional neuroanatomical levels and processes in error commissioning that are described in section A Cognitiv‘e View on Error Commission. The red lines denote feedback loops that mediate post-error neural mechanisms. **(B)** Illustration of the relative strength/stability of prefrontal representations and distractor saliency at the basal ganglia level. The maps indicate the strength/stability of the task goal representation in the prefrontal cortex (PFC) as well as the saliency of a (distractor) stimulus at the perceptual/attentional level. As outlined in the text, it is assumed that both the stability of the PFC representations and the saliency of (distracting) stimuli are commonly represented in fronto-striatal networks. The heights of the respective “blobs” in the activation map are important. In the leftmost part of the figure, distractor information is likely to be canceled out because the stability of the prefrontal representation is high. In the right-most part of the figure, the distractor is highly salient and therefore likely leads to overwriting of the task-goal representation. Note that the figure is only an illustration of the core components described in the review; the underlying computations have only the function to stipulate the main points.

Consider a typical situation that provokes response errors and is characterized by discrepancies between a desired goal state and the response that is actually executed. Figure 2 depicts a perspective about current cognitive and neurophysiological theories about how action and behavioral adaptation are implemented.

It can be assumed that task-goal representations are stored in the working memory in the prefrontal cortex (PFC) and as a copy that is likely transferred to the basal ganglia (BG) via functional prefrontal BG loops (Chudasama and Robbins, 2006). Due to this transfer, a representation of the task goal is also set up in the BG, and sensory input is simultaneously provided to the BG. These different inputs form a “map” of different neural activities that may vary in strength (Figure 1, Alternative B). However, the selected action does not only depend on the neural representations but also on those parameters of stimulus processing (Lawrence et al., 2003) that are related to differences in the saliency of stimuli.

Suppose there are two stimuli (A and B) that differ in saliency and are related to opposing actions (A = correct; B = erroneous) that are attempting gain control over behavior. Due to their different saliencies, these actions evoke correspondingly different degrees of activation in their competition with each other (Desimone and Duncan, 1995; Reynolds and Chelazzi, 2004; Knudsen, 2007). Whether stimulus A wins this perceptual competition depends on (i) the relative saliency of stimulus B and (ii) the intentional biases (i.e., top-down influences) that favor stimulus B and disfavor the processing of stimulus A (Knudsen, 2007). The net result of these perceptual competitive influences determines whether feature A or B is detected and controls behavior. If the net result favors the erroneous stimulus B, stimulus A loses the competition, is not detected and will not govern behavior (Desimone and Duncan, 1995). In addition to these factors, other factors also influencing this net-result may be related to top–down attentional biases, like previously learned stimulus-response associations or bottom–up influences on these association strength like spontaneous fluctuations and lapses in the attentional system (which is of particular importance when considering altered error processing/commission in diseases; e.g., Weissman et al., 2006; Sonuga-Barke and Castellanos, 2007; Sarter and Paolone, 2011). Thus, this process may ultimately lead to an error. We assume that the net result of these processes is also fed into fronto-striatal loops. Within the basal ganglia, an action selection mechanism operates using the principles of a “winner-takes-all” network that converges to a single winner (Bar-Gad et al., 2003). The most salient of the representations provided by the PFC, sensory signals from the primary sensory areas and efference copies of motor activity is selected. In the next step, the winning representation is fed to the motor cortex, and the response is executed. There are at least three possible constellations by which a correct task-goal representation can compete with other, error-favoring sensory inputs (Figure 1, Alternative B). If the neural activation at the BG level caused by the correct task representation is stronger than the other neural activations, the correct task representation wins, and the correct response is likely to be executed. In the opposite case of a dominant irrelevant sensory input, an erroneous response is the most likely to be provoked. However, if the activations of task representation and the sensory input are equivalent, the motor response may be selected almost randomly via a stochastic process (e.g., consider a situation in which one is uncertain about the correct response) or even activate conflicting motor responses. However, task processing does not end with the initiation of response execution because inadequate task handling (irrespective of being erroneous or unexpected) requires controlled adaptation. This control and the corresponding structures (i.e., the ACC and PFC) are the next important functional stations in this framework that should be considered. Once the response is executed, a novel action can, in principal, be executed immediately. However, if the previous response was erroneous, this processing sequence is not adequate. To adapt behavior, the previously described process can begin again but has to wait for the outcome of an evaluation that provides information about the adequacy/correctness of the executed response. At this point, the above-mentioned neuro-cognitive perspective on error commissioning connects with the established error processing models that focus on the consequences of an error and how those consequences are used to adapt behavior.

An important aspect of the above neuro-cognitive conception of error commissioning is that the likelihood of committing an error is not stable over time. Rather, the fluctuations in error likelihood are grounded in the processes discussed above. We assume that each of the above-described factors reveals a specific processing pattern over time. For prefrontal processes, this pattern is related to fluctuations in the stability of task-goal representations that might emerge because in prefrontal networks stability of information is not stable, but fluctuates. Moreover, fluctuations in the saliency of error-favoring sensory input emerge due to the ability to suppress this erroneous input and the relative saliencies of relevant and irrelevant (i.e., error-favoring) inputs. The interplay of each of these factors then determines the resulting error likelihood. This interplay likely depends on different neurobiological factors and their temporal properties. Fluctuations in error likelihood are therefore not only determined by the strengths of representations in the prefrontal networks. Rather, fluctuations in error likelihood are also influenced by mechanisms related to attentional selection and vigilance that are associated with the perceptual processing of stimuli. However, the precise contribution of each of these processes might vary considerably. To provide a simple illustration of error likelihood, only the influences of the strength of the task representation in the PFC and the saliency of the error-favoring stimulus input are depicted in Figure 1, Alternative B. The left image in Figure 1, Alternative B represents a putative case of low error likelihood at the time point of action selection. In this case, the strength of the prefrontal representation is considered to be high, and the saliency of error-favoring stimuli is considered to be low. The resulting error likelihood emerges by combining both factors. However, error likelihood can increase (Figure 1, Alternative B, middle and right); for example, if low-stability task representations coincide with a high-saliency error-favoring sensory input at the time of action selection, a high likelihood of error will result. Indeed, the error likelihood at the time of action selection can remain at a fairly low level for some period of time and can also remain at a fairly high level for some period of time. Obviously, the durations of the time periods in which the fluctuation of the error likelihood is low depend on the strength of the task-goal representation and the saliency of the error-favoring sensory input.

### Details on the neuro-cognitive basis of error-commissioning

Having described factors that may play a role in error commissioning, several established conceptions appear to play roles in a comprehensive account leading to an understanding of the principles resulting in response errors. The processes described above can be understood in terms of the core ideas of reinforcement learning theory (Holroyd and Coles, 2002) and conceptions proposing that the basal ganglia play an important role in action selection (e.g., Bar-Gad et al., 2003; Gurney et al., 2004b; Maia and Frank, 2011). Moreover, assumptions of the dual-state theory of dopamine function (Seamans and Yang, 2004; Durstewitz and Seamans, 2008) and the guided activation theory (Miller, 2000; Miller and Cohen, 2001) seem to be important when trying to examine the mechanisms that lead to an error, i.e., the investigation of error *commissioning*. This is because these theories deal with questions how representations stored in prefrontal networks are maintained and used to guide action selection. Obviously, these are important to consider the processes that lead to an error. In particular, in tasks that require continuous and rapid processing of stimuli, errors emerge due to attentional lapses or even conflicts in stimulus processing. Thus, aspects of attentional selection processes also have to be considered. In this regard the “biased competition model” of attention (Desimone and Duncan, 1995) may provide useful grounds. However, the investigation of the processing of information and action selection (or decision) from a neurophysiological perspective should not ignore the heuristic value of the existing well-established cognitive approaches because these provide a solid theoretical basis regarding how information is processed and how errors might emerge. Thus, it appears at hand to combine different theoretical approaches within the field of neuroscience, with established psychological models such as the “drift diffusion model” (DDM; Ratcliff, 1978, 1979, 1980, 2006; Ratcliff and Rouder, 1998; Vandekerckhove and Tuerlinckx, 2007; Ratcliff and McKoon, 2008) to approach the question how errors are committed. In the forthcoming we detail what how these factors may influence error commissioning and what aspect are necessary to consider in future computational models on error commissioning.

#### From prefrontal networks to attentional selection

Central to the above-suggested integration of different concepts are assumptions about the stability of information in the prefrontal cortex (PFC). In nearly every daily situation people have a goal that they try to fulfill. Often, this goal is the establishment of proper stimulus-response mappings in terms of the defined task at hand; e.g., “*Respond with the left hand when stimulus X is present, and respond with the right hand when stimulus Y is present*.” Such task-goal representations are stored in working memory buffers within the prefrontal cortex (PFC) (e.g., Jonides et al., 2008). The most established assumption about the function of the PFC networks is that they hold and manipulate information (task-goal representations) for future use via persistent activity states (e.g., Seamans and Yang, 2004). Within the PFC, dopamine (DA) influx may serve as a gating signal that instructs the network when to maintain a given activity state (Miller, 2000). The neuromodulatory effects of dopamine may strengthen current representations and protect them against interference due to disruption by irrelevant distracting information (Miller, 2000). According to the “dual-state theory of dopamine function,” whether new information can easily access working memory buffers (state 1) or current representations are maintained and stabilized within prefrontal networks (state 2; Seamans and Yang, 2004; Durstewitz and Seamans, 2008) appears to depend on the state of the dopaminergic system.

In the first state (state 1), D2-receptor-mediated neural transmission predominates and allows multiple inputs to access working memory buffers. In contrast, the second state (state 2) is dominated by D1-receptor-related neural transmission. In this state, the working memory buffers are relatively closed, but information held within these buffers is more stable and controls the output of prefrontal networks (Miller, 2000; Seamans and Yang, 2004). It may be speculated that these different states serve different brain functions in relation to performance monitoring. Recent evidence has shown that D2-receptor mediated neural transmission, though less important in the prefrontal cortex, is associated with exploitative learning that adjusts response times as a function of positive and negative outcomes (Frank et al., 2009), which suggests that D2-receptor-mediated neural transmission is of particular importance for processes that are related to error commission and monitoring. However, the D1 and D2 states fluctuate spontaneously and are organized in an antagonistic fashion. During phases in which the D1-state dominates, D2-receptor-related neural transmission is less active, and vice versa. In the mechanism that mediates the transitions between the D1 and D2 states, it is assumed that GABAergic and NMDA processes play important roles (Seamans and Yang, 2004; Durstewitz and Seamans, 2008). It is assumed that increases in D1-receptor activation initially augment the robustness of representations within the PFC (Seamans and Yang, 2004). However, further increases above an optimal level reverse these effects and shift the system away from robustness. This process leads to changes in the mode of the cognitive processes occurring in the PFC. The dual-state theory of the dopamine system is not the only approach to assume the existence of dynamic gating of representations in the PFC (Hazy et al., 2010; O'Reilly et al., 2010). As illustrated in the work of Dayan (2008, 2007), several approaches on PFC functioning involve switching between network states that either process information according to a habit that is inflexible or more goal-directed and rule-governed states that enable the flexible processing of task goals. In this regard, the biophysical properties of the PFC that determine the stability of information and other models of prefrontal cortical functioning reflect the assumption of fluctuating states and the stability of task goals in the PFC and, hence, the essential basis of the assumption of differences in error likelihood over time.

However, the above-described mechanisms related to task goal representations in the PFC have important consequences for information processing in other brain areas. This idea constitutes the core of the “guided activation theory” (Miller, 2000; Miller and Cohen, 2001). This theory proposes that representations of task-specific rules stored in PFC networks form attentional templates and goals (see also: Wood and Grafman, 2003) that are needed to enable goal-directed behavior (Miller, 2000). This task information enables the PFC to control processing in other brain systems and direct that processing toward task-relevant information (Miller, 2000). The aggregate effect of these bias signals is to guide the flow of neural activity along pathways that establish the proper mappings between the inputs, internal states and outputs that are needed to (correctly) perform a given task (Miller and Cohen, 2001). This control is particularly important whenever stimuli are ambiguous, i.e., when stimuli activate more than one input representation (Miller and Cohen, 2001). From this perspective, the constellation of PFC biases can be viewed as the neural implementation of rules or goals depending on the target of their biasing influences. If these “bias signals” affect sensory modalities, this can affect the mechanisms of attentional control and thereby affect another major factor that may influence error commissioning: attentional selection processes.

Frontal regions, including the anterior cingulate (Cabeza and Nyberg, 2000; Lawrence et al., 2003), the right frontal cortex (middle and inferior frontal gyrus) and bilateral parietal regions (i.e., inferior parietal cortex), play a role in maintaining and controlling attention over time (Posner and Petersen, 1990; Coull, 1998). They are the source of the biasing influences on sensory processing (Knudsen, 2007) that can reduce the saliency of the error-favoring stimuli. To the extent that biasing influences on attention depend on representations in the prefrontal networks (Desimone and Duncan, 1995; Knudsen, 2007), these mechanisms may also be affected by the fluctuations in prefrontal networks described above. As an effect that these influences are not always able to bias attentional selection toward desired task goals, which will, in addition to influences related to the relative saliency of error-leading stimuli, determine error likelihood. The “biased competition theory of attention” (Knudsen, 2007; Desimone and Duncan, 1995) assumes that the outcome of perceptual competition is determined by the saliency of the stimuli (Beste et al., 2011b, 2012a; Beste and Dinse, 2013) and intentional biases (top-down influences). The net result of these perceptual competitive influences determines whether feature A or B is detected and controls behavior (Sänger and Wascher, 2011; Labrenz et al., 2012). When the net result favors the erroneous stimulus B, stimulus A loses the competition, is not detected and does not govern behavior (Desimone and Duncan, 1995), which may ultimately lead to an error. Thus, fluctuations in the stability of the task goal representation in the PFC affect attentional selection processes and are therefore another major property that determines the likelihood of committing an error. It is simply the lack of biasing influences on attentional selection processes that further contributes to the likelihood of committing an error. Both aspects of error commissioning, i.e., the stability of the task goal representation and attentional selection processes, are interrelated determinants of error commissioning.

#### Convergent input to the basal ganglia and the “winner-take-all principle”

However, functioning of the prefrontal cortex cannot be understood without referring to the basal ganglia, as these structures are well known to from closed functional loops (e.g., Chudasama and Robbins, 2006). Moreover, when considering the possible importance of the dopamine system for fluctuations in error likelihood and hence mechanisms of error commission, the basal ganglia need also be taken into account. The basal ganglia are important when considering the gating functions of the prefrontal cortex (O'Reilly et al., 2010) that are related to shifts in the processing modes of task goal representations in prefrontal cortical networks. However, to fulfill this requirement, the basal ganglia must serve as a “hub region” that receives information from the PFC and is modulated by processes occurring in the sensory modalities. It is well established that the PFC is closely connected to the basal ganglia via distinct functional loops (Chudasama and Robbins, 2006). Several theoretical accounts suggest that the basal ganglia are central to response selection mechanisms (Redgrave et al., 1999; Gurney et al., 2001, 2004a; Humphries and Gurney, 2002; Humphries et al., 2006; Maia and Frank, 2011). Conceptions that stress the importance of basal ganglia structures in action selection and control propose that the selection of actions (motor commands) depends on the relative salience of competing actions and that the most salient competitor wins this selection process (Redgrave et al., 1999, 2011). Action selection should be terminated when it has been successful or if it proves to be ineffective or erroneous. The selection of a correct action may also be “interrupted” by a competitor that is relatively more salient than the desired action (Redgrave et al., 1999) because action selection at the level of the basal ganglia is described by a “winner-take-all” (WTA) mechanism (Kropotov and Etlinger, 1999; Redgrave et al., 1999; Plenz, 2003). Following selection, the winning outcome may begin to reduce the salience of its predisposing conditions (as these become partially fulfilled). When the salience of a selected action falls below that of a close competitor, this competitive action will be executed (Redgrave et al., 1999). This WTA mechanism that mediates the selection of actions is implemented using a neural network that converges to a single winner (Bar-Gad et al., 2003). In biologically constrained computational models (Gurney et al., 2004b), these mechanisms are modeled as the action of striatal medium spiny neurons (MSNs). The MSNs are strongly modulated by the dopaminergic system via dopamine D1 and D2 receptors (overview: Surmeier et al., 2010). The dense network of inhibitory connections between MSNs is assumed to inhibit neighboring neurons and thereby maintain the activity of only a single neuron. The selection process takes place within the striatum, and the chosen action is then conveyed to the output layer of the basal ganglia and subsequently fed back to the cortex where the selected response is executed (Bar-Gad et al., 2003). Because bias signals affect response execution processes (Miller and Cohen, 2001), it is reasonable to assume that representations or contextual inputs may influence basal ganglia processes and, hence, WTA (for a review see: Samejima et al., 2005; Redgrave and Gurney, 2006). This assumption is even more likely because the functioning of prefrontal cortical areas cannot be understood in isolation from the modulatory influences of the basal ganglia (Parvizi, 2009). The ultimate effect of these bias signals is the re-weighting of the relative influences of goal-directed and error-favoring representations in the basal ganglia. The basal ganglia WTA network “decides” in favor of the activation with the strongest representation. In turn, the basal ganglia WTA mechanism most likely selects another (possibly correct) action simply because the relative weights favor that action. This re-weighting of task representations at the level of the basal ganglia may be attributable to one or both of the following processes:

- the correct task representation is strengthened in the prefrontal cortical networks;
- the sensitivity of the neural structures of the error-favoring information is reduced.

The above-mentioned (“re”)-strengthening of task representations in the PFC by dopaminergic influx (Miller, 2000) is necessary because the error may partly have occurred due to weaker task-goal representations, which might ultimately lead to a disadvantageous pattern of activations in the basal ganglia that may foster the occurrence of an error, particularly when a distracting, error-favoring sensory input is concomitantly present.

Similar to prefrontal representations, basic visual inputs enter the basal ganglia networks (Hikosaka, 1989; Hikosaka and Wurtz, 1989; Silkis, 2000; Coizet et al., 2007; Redgrave et al., 2011). A growing body of evidence suggests that a sub-cortical structure in the dorsal midbrain (i.e., the superior colliculus) is the most likely source of the early visual input to dopaminergic neurons (Redgrave and Gurney, 2006; Silkis, 2000). This subcortical pathway has been substantiated through electrophysiological studies in rats, cats and monkeys (Comoli et al., 2003; McHaffie et al., 2006). Thus, several cortical structures provide convergent input to the basal ganglia, and this input consists of (i) a set of stimuli, (ii) a task set that has been established in the PFC, and (iii) a corresponding efference copy. This information basically provides the basis for action selection in striatal networks (Bar-Gad et al., 2003; Redgrave and Gurney, 2006).

As originally conceptualized by Redgrave et al. (1999), the idea of a WTA mechanism is also central to other computational models of response selection in the basal ganglia. The model of Bar-Gad et al. (2003) conceives of MSNs as central to the comparison of different response options and, hence, the selection between those options. Similar assumptions have been put forward in the den model of Plenz (2003) and the model of Plenz and Kitai (1998). Moreover, other recent models (e.g., Humphries et al., 2006) assume that the basal ganglia perform response selection processes via a restrictive mechanism; however, these models assume that an important property of the regulation of action selection in the basal ganglia is the dopaminergic system. Humphries et al. (2006) assumed that response selection and control are mediated via different dopaminergic receptor systems. This assumption is also implemented in other models of the basal ganglia (e.g., Maia and Frank, 2011; Wiecki and Frank, 2013) in which the distinctions between dopaminergic subsystems include Go- and Nogo-neuron populations. These latter models developed by Frank et al. therefore primarily address response selection in terms of response inhibition mechanisms (Wiecki and Frank, 2013). However, the models can also be applied to two-choice decision-making and response selection processes, which are the focus of the model of Humphries et al. (2006). These other conceptions of action selection in the basal ganglia are not at odds with the WTA network upon which our error-commissioning model is based because the efficiency of the WTA mechanism is modulated by dopaminergic signaling (Gurney et al., 2004a; Humphries et al., 2006).

Supposed roles of the BG and the PFC in error commission may be linked to drift-diffusion models (DDM). The model developed by Frank (2005) suggests that following stimulus presentation, the medial PFC generates possible actions with different probabilities of execution that depend on the specific stimulus (Cavanagh et al., 2011). This notion is in line with the finding that a correlate of response monitoring, i.e., the CRN, varies as a function of S–R mapping (refer Box 2): the CRN and thus PFC functions appear to be attenuated if the probability of a certain response is reduced because, prior to response selection, a defined visual spatial stimulus position is also inhibited (Hoffmann and Wascher, 2012). However, it can be assumed that if there is a response conflict, the medial PFC-STN network should increase the decision threshold (i.e., the boundary separation) to enable the cortico-striatal network to assess the reward values of the response alternatives (Cavanagh et al., 2011). Cavanagh et al. investigated this assumption via integration of the core ideas of the BG model of Frank (2005, 2006) and the DDM. More specifically, they investigated whether the subthalamic nucleus (STN), as important basal ganglia structure receiving input from the frontal cortex, has an inhibitory influence during decision conflict. They found that trial-to-trial medial PFC activity, as measured based on EEG theta power, is correlated with the threshold for evidence accumulation. This relationship is modulated by conflict. Besides this correlative finding they found that deep-brain stimulation of the STN in Parkinson's patients strongly modulates this relationship. Further corroborating the relevance of basal ganglia processes, are studies by Forstmann et al. (2008, 2010). These studies show that decision processes, modeled by DDM-like processes are strongly related to basal ganglia processes (Forstmann et al., 2008). Importantly, activation in cortico-striatal networks and especially the subthalamic nucleus, considered to be important for processes of error commission (refer sections above), have been shown to directly predict the modulation of decision processes at the behavioral level in choice-response tasks (Forstmann et al., 2010). Due to the strong interrelation of the DDM with neurobiological processes of the basal ganglia, the DDM provides opportunities to test a key assumption of error commission on a behavioral level, i.e., the modulation of error likelihood. The DDM allows a quantification of the parameters that reflect inter-trial fluctuations in decision processes. These fluctuations are captured by the variability in the drift rate (e.g., variability due to fluctuations in attention), starting point (e.g., due to online reward or punishment) and the non-decisional parameter. Apparently, there is a close link between the DDM and the error commissioning framework proposed herein; i.e. according to the framework presented, fluctuations in error likelihood are determined by the strengths of the representations in prefrontal networks, which are akin to the boundary separation and starting point variabilities in the DDM. Moreover, the mechanisms associated with attentional selection are conceptually similar to the drift rate parameter proposed in the DDM. Aspects of vigilance are closely related to the perceptual processing of stimuli, which is akin to the non-decisional parameter and its variability. Accordingly, if these processes/structures are manipulated, one would expect variations in the corresponding DDM parameters and, hence, changes in the fluctuations in error likelihood, as outlined at the end of this article. It should be noted that the neurobiological mechanisms modulating fronto-striatal networks described above are not exhaustive. There are many other modulators affecting processing in prefrontal networks.

#### Timing constraints in error-commissioning

It can be assumed that the basal ganglia and the action selection processes mediated via these structures play an important role when considering factors possibly important for the commissioning of errors. Basically, the basal ganglia can be conceptualized as a “hub region” that receives different types of information. These inputs influence action selection, and hence, the mechanisms of error commissioning. It is therefore critical that the basal ganglia, as a “hub region,” receive all of this information in a timely manner.

Another aspect is the stability of task-goal representations in prefrontal structures, which is determined by dopaminergic signaling via dopamine D1 and D2 receptors. A major problem in cognitive control models is generally that the dopamine system is considered to be too slow to support the rapid processes of action selection that ultimately lead to erroneous responses. Thus, Jocham and Ullsperger (2009) proposed that error monitoring and the behavioral adaptation processes that follow an error, such as those related to the ERN, are mediated via slower dopamine responses after the error monitoring system has been activated through other (faster) neurotransmitter systems.

However, it can be assumed that the stabilities of task-goal representations are dependent on the properties of the dopaminergic system, which have been shown to exert long latency responses in prefrontal structures that last several seconds (e.g., Robinson et al., 2003; Seamans and Yang, 2004; Heien et al., 2005). However, it is not clear, whether the effects of prefrontal processes on striatal processes are mediated by the dopaminergic system *per se*: cortico-striatal synapses are glutamatergic in nature (e.g., Bolam et al., 2000), and it has recently been shown that striatal states can reliably be changed by these glutamatergic inputs and influence action selection processes (e.g., Tomkins et al., 2014). Indeed, it is likely that that changes in the stabilities of task-goal representations in prefrontal networks affect the striatal mechanisms of action control via glutamatergic projections. In addition to these “contextual” influences, “sensory” influences also modulate striatal structures. As outlined above, these sensory influences likely enter striatal structures (for a review, see Redgrave et al., 2011) and use short-latency dopamine signals. This short-latency component of the visual input to DA neurons derives from subcortical visual processing regions in the superior colliculus in the midbrain (e.g., McHaffie et al., 2006; May et al., 2009), and it has been shown that sensory processing in the cerebral cortex can drive phasic responses in DA neurons (for a review, see Redgrave et al., 2011). These findings show that dopaminergic neurons are active *prior* to behavioral responses. The dopaminergic system may thus play a role in error commissioning either at the level of short latency dopaminergic signaling or at the level of task-goal representations in the prefrontal cortex. However, the latter, slow dopaminergic influences at the neocortical level, might not be the only dominant mechanism.

#### Error processing

The review above describes what neuronal components are necessary to consider when being interested in error commissioning. Mechanisms of error processing or more specifically adaptation are not addressed, because these processes have been formalized in well-established models and it has been shown that the ACC plays an important role (e.g., Holroyd and Coles, 2002). An overview of theories on error processing can be found in Box 1.

Bush et al. (2000) distinguished the ACC regions that are involved in “cognitive” and “emotional processing”. These authors stated that “cognitive” processing is related to the 32′, 24c′, 24b′m, and 24a′ subregions and, thus, to the dorsal part of the ACC (dACC). In contrast, emotional processing is due to regions 32, 24c, 24b, 24a, and 25 and therefore to the ventral part of the ACC (vACC). The functions of the ACC range from very basic (homeostatic) to more complex social-cognitive functions. In general, one can assume that the core function of the ACC is to establish the mobilization required to cope with cognitive and, as recently described, emotional and social demands (Mayberg et al., 1999; Elliott et al., 2000; Paus, 2001; Phillips et al., 2003) to achieve cognitive control (Koban and Pourtois, 2014). Thus, the ACC plays a role in the processing of tasks that require increasing cognitive effort and control and the management of responses when faced with conflicting demands (Luu et al., 2003; Fan et al., 2008). Furthermore, the ACC is a central structure involved in determining how to act in a goal-directed manner and is central to inhibitory control (Braver et al., 2001; Bari and Robbins, 2013), reward responses and their modulation (Amiez et al., 2005) as well as the top-down influence on primary sensory processes (Crottaz-Herbette and Menon, 2006). Together, these results suggest that the ACC is well suited for comparing the results of action selection to the task-goal representation (Bush et al., 2000). This assumption is further supported by the connections of the ACC with even distant regions in the dorsolateral prefrontal cortex (DLPFC), which are important for working memory functions (Paus, 2001). In combination with the connections with the motor areas, this extensive connectivity of the ACC with the lateral regions of the PFC may provide the basis for an interchange between the cognitive and motor systems (Paus, 2001) that is necessary to perform evaluator functions and to provide information that can be used for behavioral adaptation (Paus, 2001). This role is stressed in recent views regarding the functions of the ACC indicating that the diversity of the functions mediated by the ACC can be understood in terms of a single function, i.e., the allocation of control based on an evaluation of the expected value of control (Shenhav et al., 2013). In this sense, this error signal may function as a biasing signal (Miller and Cohen, 2001) that instructs the prefrontal networks when to maintain a given activity state (representation) (Miller, 2000; Shenhav et al., 2013). This error biasing signal has been demonstrated using fMRI and EEG data (Debener et al., 2005; Hoffmann et al., 2014). The error monitoring signal can therefore be employed to strengthen representations in the prefrontal networks. Thus, the error signal closes the circle and influences the processes described above that lead to the commissioning of an error. A brief description of the properties of the error processing signal is given in Box 2.

In this sense, the ACC may play a central role not only in the processing of errors, but also in processes preceding erroneous or successful response selection. A recent integrative account, the “expected value of control (EVC)” model (Shenhav et al., 2013) proposes that the (dorsal) ACC uses information from the current state (i.e., information of task demands, processing capacity etc.) and expected value of the outcomes to determine how much cognitive control is invested in a given task (Shenhav et al., 2013). Critically for its potential role in processes leading to errors are findings showing that above-mentioned processes supposed to be important elements in the processes that lead to an error (i.e., strength of task goal representation, attentional biases, conflict monitoring and cognitive control) are all related to the functioning of the (dorsal) ACC: the ACC differentiates task representations and response rules (e.g., Dixon and Christoff, 2012), specific actions and task sets (Hampton and O'Doherty, 2007; Haynes et al., 2007). For an excellent overview of the ACC's functions refer to Shenhav et al. (2013). In sum, there is hence ample evidence to that the ACC is involved in processes that we are proposing to be central for the understanding of how errors emerge. The ACC may thus not only be seen as an element in error processing, but also as an important entity in mechanisms that may ultimately contribute to the occurrence of errors.

## Concluding remarks and outlook

The theoretical integration outlined above details the possible neurophysiological mechanisms that may be crucial for the commission and detection of errors and behavioral adaptation. We suggest that error likelihood is a dynamic process that depends on the functional and temporal properties of brain regions that influence prefrontal-basal ganglia networks. Unlike other reviews of cognitive control and error processing, the review presented here describes the possible mechanisms that lead to the commission of an error and the identities of the functional neuroanatomical and neurobiological systems that may play roles in this process. Importantly, it is proposed that error likelihood is not stable but fluctuates over time. The “rhythm” of fluctuation is most likely determined by different neurobiological properties of the outlined factors within each of the considered neuro-functional systems that must be taken into account. Thus, a major challenge for future research will be the identification of the neurobiological factors (and their interactions) that determine the functional and temporal properties of the brain regions that ultimately modulate the likelihood of response errors over time. This will be a necessary requirement before formal computational models of error commissioning can be set up, because otherwise too many factors might be modeled making possible computational approaches too complex and thus quite difficult to be empirically validated. Several starting points are available for the initiation of an investigation of the determinants of fluctuations in error likelihood and hence the neural mechanisms underlying error commissioning. However, it is necessary to use behavioral parameters and psychological frameworks that allow the characterisation of response selection processes to pursue the dynamics of error likelihood from a neuroscientific perspective. In this regard, it will be necessary to include components of drift diffusion models (DDMs; Ratcliff, 1978, 2006; Vandekerckhove and Tuerlinckx, 2007; Ratcliff and McKoon, 2008).

### Starting point 1

As outlined in the above sections, a major component determining error likelihood is the stability of the task goal representations in the prefrontal cortex, which are largely determined by dopaminergic neural transmission via different receptors. The stability of task goal representations may bias subsequent processing of incoming information. The strength of task goal information may therefore have also an influence on how much information is needed to reach a (correct or erroneous) decision. When referring to the DDM this is reflected in the “starting point” parameter (*z*). The starting point (*z*) describes the a priori bias associated with one of the choices. Pharmacological studies in humans and animals may specifically target dopamine D1 and D2 receptor neural transmission to modulate fluctuations in error likelihood. Similarly, molecular genetic studies examining the relevance of different single nucleotide polymorphisms that affect dopaminergic neural transmission will be of interest. However, the use of pharmacological approaches will enable the determination of dose-response functions that efficiently modulate the fluctuations in error likelihood over time. It remains to be determined whether dopamine D1 or D2 receptor-mediated neural transmission will be more important in modulating error likelihood. Regardless, agonistic modulation of dopaminergic D2 receptors should induce stronger fluctuations in error likelihood than agonistic modulations of the D1 receptor system because the task goal representations that guide response selection are less stable. However, regarding the relevance of the prefrontal dopaminergic system, it will be of great interest to examine psychiatric and neurological diseases that affect the dopaminergic system, such as schizophrenia and Parkinson's disease. Several lines of research suggest that the processing of errors is altered in these diseases. Research on fluctuations in error likelihood will broaden this perspective in such a manner that different diseases that affect the dopaminergic systems may be viewed not only as “error-processing disorders” but also as disorders of the processes that lead to errors. By modulating the dopaminergic system and thereby a parameter that may affect task goal representations, it is possible that parameter (*z*) of the DDM is modulated.

Notwithstanding the relevance of the dopaminergic system as a starting point for investigations of fluctuations in error likelihood, the striatal GABAergic medium spiny neuron system is also relevant. One option for investigating this functional relevance may be targeted pharmacological manipulation of the striatal GABAergic system using muscimol in animal experiments. In humans, another option may be to investigate diseases that affect the functioning of striatal medium spiny neurons in combination with dopaminergic dysfunctions. One possible way to complete the link to the relevance of the dopaminergic system together with structural basal ganglia changes is investigation of neurodegenerative diseases such as Parkinson's or Huntington's disease. However, the examination of rare disease models that lack dysfunctions of the dopaminergic system but exhibit circumscribed dysfunctions of basal ganglia structures may enable confounds due to alterations of the dopaminergic system and changes in the structural basal ganglia to be avoided. For example, benign hereditary chorea (BHC) may be used as a model disease (e.g., Beste and Saft, 2013, 2014). Additionally, advances in GABAergic magnetic resonance spectroscopy related to the neurobiochemical properties of striatal structures and cognitive control functions (e.g., Yildiz et al., 2014) and, hence, the fluctuation in error likelihood may also be of relevance from a systems perspective and for examination of the striatal GABAergic system. As regards the potential role of the GABAergic system in processes leading to an error and with respect to the DDM it is possible that the GABAergic may affect both, the parameter (*z*) and the drift rate. The latter describes how much information is needed for a decision. This dual role of the GABAergic system for parameters (*z*) and the drift is plausible since the GABAergic is central to the comparison of different response options. This comparison process needs information, whose accumulation is reflected in the drift rate. On the other hand the GABAergic system is assumed to play a role in the maintenance of task goal. There the GABAergic system may also affect parameter (*z*) in the DDM.

### Starting point 2

In addition to these neurobiochemical and disease approaches for examining the functional neuroanatomical constraints on fluctuations in error likelihood, another important starting point will be the manipulation of information processing at the cortical level within in the prefrontal cortex and sensory processing areas. Such manipulations are important because error likelihood may be at least partially determined by competitive attentional selection processes. Perturbing information processing in these structures using transcranial magnetic stimulation (TMS) or perceptual learning mechanisms (Beste et al., 2011b; Beste and Dinse, 2013) will help to elucidate the relevance of these functional neuroanatomical structures in the dynamics of error commissioning. Using such an approach it should be possible to elucidate the relative contribution of different parameters of the DDM in close relation to circumscribed functional neuroanatomical regions. As outlined in the previous section, processes from the perceptual level to the level of task-goal representations may be important in the cascade of error commission. It is possible that by targeting sensory processing (areas), the drift rate as indicator of the speed of information accumulation is affected. Perturbing information processing in prefrontal structures may, on the other hand, be more relevant to the modulation of the boundary separation parameter (*a*) and starting point (*z*), as these parameters are both reflecting aspects/consequences of task goals.

Clearly, the above-mentioned exemplary approaches (starting points) initially have to be applied with a focus on specific components of the described error-commissioning network. Each of these components contributes to the fluctuation in error likelihood, and the contribution of each component to the fluctuation in error likelihood may be described as a function of time in mathematical terms. As in the excerpts described above, it might be possible to derive neurobiologically constrained “functions” that describe the dynamics of cognitive processing in different brain regions and cognitive systems using targeted approaches. Hence the individual contributions of these processes to the fluctuation in error likelihood can be described. It remains to be determined which and how many of these functions will be found to be important for description of the fluctuation in error likelihood over time. However, as error commissioning and the fluctuation in error likelihood are conceptualized as a the interplay of different functional neurobiological and neuroanatomical systems, a coherent view of the determinants of error commissioning will require approaches that examine previously identified functions that simultaneously determine fluctuations in error likelihood. Mathematically speaking, error likelihood may be described as the folding of each of these contributing “sub-functions” that partially determine the fluctuations in error likelihood. However, it remains to be seen how much variance in the fluctuations in error likelihood will be explained by each of these functions. Using approaches that examine the influences of various factors that simultaneously determine the fluctuations in error likelihood, it will be possible to determine which aspects contribute most strongly to the fluctuations in error likelihood. Using a combined approach, it will, in the long term, be possible to examine how many aspects of information processing need to be monitored to predict the occurrence of an error to a reasonable degree. This investigation is tantamount to the question of which neurobiological components are most important for the fluctuations in error likelihood. Most likely, the different components need to be weighted according to their relative importance in determining the fluctuation in error likelihood, and this relative importance may be further subject to inter-individual differences that are grounded in inter-individual variations in the neurobiological factors that determine the processing of cognitive systems that are important for error commissioning and the fluctuation of error likelihood.

### Conflict of interest statement

The authors declare that the research was conducted in the absence of any commercial or financial relationships that could be construed as a potential conflict of interest.
